# An exploration of the statutory Healthy Start vitamin supplementation scheme in North West England

**DOI:** 10.1186/s12889-022-12704-0

**Published:** 2022-02-24

**Authors:** May Moonan, Gillian Maudsley, Barbara Hanratty, Margaret Whitehead

**Affiliations:** 1 Department of Public Health and Policy, The University of Liverpool, Liverpool, UK; 2 Present Address: Warrington Hospital (Kendrick Wing), Warrington, UK; 3 Department of Public Health, Policy, and Systems, The University of Liverpool, Liverpool, UK; 4 Population Health Sciences Institute, Newcastle University, Newcastle upon Tyne, UK

**Keywords:** Child–preschool, England Healthy Start, Food subsidy, Food voucher, Health inequalities, Mothers, Nutrition, Poverty, Universal and targeted services, Vitamins

## Abstract

**Background:**

Government nutritional welfare support from the English ‘Healthy Start’ scheme is targeted at low-income pregnant women and preschool children, but take-up of its free food vouchers is much better than its free vitamin vouchers. While universal implementation probably requires a more extensive scheme to be cost-effective, the everyday experience of different ways of receiving or facilitating Healthy Start, especially via children’s centres, also requires further evidence. This study therefore aimed to explore (in the context of low take-up levels) perceptions of mothers, health professionals, and commissioners about Healthy Start vitamin and food voucher take-up and compare experiences in a targeted and a universal implementation-area for those vitamins.

**Methods:**

Informed by quantitative analysis of take-up data, qualitative analysis focused on 42 semi-structured interviews with potentially eligible mothers and healthcare staff (and commissioners), purposively sampled via children’s centres in a similarly deprived universal and a targeted implementation-area of North West England.

**Results:**

While good food voucher take-up appeared to relate to clear presentation, messaging, practicality, and monetary (albeit low) value, poor vitamin take-up appeared to relate to overcomplicated procedures and overreliance on underfunded centres, organizational goodwill, and families’ resilience.

**Conclusion:**

Higher ‘universal’ vitamin take-up may well have reflected fewer barriers when it became everyone’s business to be vitamin-aware. *Substantive* Healthy Start reform in England (not just cosmetic tinkering) is long overdue. Our study highlights that ‘policy, politics, and problem’ should be aligned to reach considerable unmet need.

**Supplementary Information:**

The online version contains supplementary material available at 10.1186/s12889-022-12704-0.

## What is known about the topic

- Take-up of free Healthy Start vitamin vouchers targeted to low-income pregnant women and preschool children in England is very low, despite good food voucher take-up.
- Suggested barriers include low maternal awareness and motivation, poor access, and health professionals’ mixed messaging or disengagement, but universal provision might improve access.
- Qualitative evidence is limited about receiving or facilitating these vitamin vouchers, particularly targeted vs universal access, via children’s centres.

## What this paper adds

- Improving presentation, messaging, and practicality of vitamin vouchers may well improve take-up.
- Poor vitamin take-up may well reflect overcomplicated procedures while relying on underfunded centres, organizational goodwill, and families’ resilience.
- Higher ‘universal’ vitamin take-up may well reflect that being vitamin-aware becomes everyone’s business.

## Background

As health inequalities continue to widen in the United Kingdom (UK), government nutritional welfare support from the ‘Healthy Start’ scheme for pregnant women and preschool children remains crucial to health improvement. Poverty-related food insecurity has increased over the last decade [1], with low-income households facing complex cost, access, and availability barriers to healthy eating [1, 2].

Sure Start ‘children’s centres’ for preschool child and family support have facilitated Healthy Start implementation in England, including maternal information and advice. Children’s centres have been particularly effective in disadvantaged areas [3] but have progressively dwindled [1, 3] since ringfenced funding ended (2011) exposing them to government austerity.

In England, Marmot et al.’s 2020 ten-year review of health equity [1] has recommended re-investing in early years’ services, including children’s centres. In the COVID-19 pandemic, children’s centres have helped in referring low-income families for foodbank support and in distributing food or vouchers, when schools are closed, to children eligible for free school meals [4, 5].

### The Healthy Start scheme

In 2006, Healthy Start replaced the Welfare Foods Scheme (established during wartime rationing in 1941, complementing the 1940 National Milk Scheme) to improve maternal and child health. Welfare Foods started as universal provision but was later subsidized and then ‘targeted means-tested’ like Healthy Start. The first major scientific review of Welfare Foods recommended urgent improvement in vitamin distribution and uptake [6].

From the outset, entitled low-income families with children under 4 (under-4 s), low-income pregnant women, and pregnant females under 18 (under-18 s) received monetary Healthy Start ‘food vouchers’ by post. These paid for cow’s milk, infant formula milk, fruit, or vegetables at registered retailers. The food vouchers may have improved maternal nutrition more than Welfare Foods [7–9]. They may well promote fruit and vegetable consumption [10, 11] although evidence is conflicting [12]. Additional ‘vitamin vouchers’ were exchanged for Government-commissioned children’s drops (6–48 months: vitamins A, C, D) and pregnant and lactating women’s tablets (folic acid; vitamins C, D). Locally arranged vitamin distribution-points included children’s centres and community health clinics.

There was initial concern about the Healthy Start design and implementation for nutritional support [13, 14]. Furthermore, piloting had revealed [15] ^**(p2)**^:

*“absence of leadership and support from senior management, and no coherent strategy for Healthy Start... Health professionals at all levels continue to work in silos, with little cross working.”*

### Healthy Start vitamins in this last decade

In February 2012, the four UK chief medical officers (CMOs) were concerned about vitamin D deficiency in at-risk groups—pregnant and breastfeeding women, under-5 s, 65-years-and over, and people with insufficient sun exposure. They reminded primary care health professionals that Healthy Start vitamins included vitamin D and were crucial for the public’s health [16], but low take-up needed tackling [7, 16, 17]. By 2016, the Scientific Advisory Committee on Nutrition (SACN) [18] was recommending whole-population vitamin D supplementation for musculoskeletal health (regarding rickets, osteomalacia, falls, and muscle strength and function). By January 2021, in recognition of more time indoors during the pandemic, clinically extremely vulnerable people and care home residents were receiving free vitamin D supplementation [19].

England’s CMO did contemplate making Healthy Start vitamin provision universal [20]. In 2012, Moy et al. reported observational evidence from Birmingham (high proportion of high-risk ethnic minorities) suggesting that changing from targeted to universal Healthy Start vitamins could reduce symptomatic vitamin D deficiency and increase public awareness about vitamin D [21]. This was despite vitamin take-up only increasing to 17% of pregnant women and preschool children. The only previous similar evidence involved Glasgow’s vitamin D supplementation for British Asian children, which statistically significantly reduced rickets [22].

Evidence is limited but suggests that low Healthy Start vitamin take-up is associated with: mixed messages about early years’ vitamin D requirements [23]; health professionals not promoting Healthy Start [24, 25]; low parental awareness about vitamin importance [21, 24] and the Healthy Start programme and vitamins [25, 26]; low maternal motivation to use vitamins [24]; poor vitamin access [24, 25]; and obstructive application procedures and logistically complicated distribution [23–25].

Calls for universal implementation [23] require further evidence from everyday experience of receiving or facilitating Heathy Start, especially via children’s centres. This study therefore aimed to explore (in the context of low take-up levels) perceptions of mothers, health professionals, and commissioners about Healthy Start vitamin and food voucher take-up and compare experiences in a targeted and a universal implementation-area for those vitamins.

## Methods

### Design and governance

Within the pragmatism paradigm, this sequential explanatory mixed-methods study (labelled as per Ivankova et al. [27]) comprised qualitative analysis of 42 semi-structured interviews with mothers and professionals, about Healthy Start voucher take-up, informed by quantitative analysis (Additional file 1). The latter used the percentage voucher ‘take-up’ extracted from quarterly data around the time of the interviews, requested from the Healthy Start Issuing Unit (HSIU) (Department of Health) [28]. Interviews were in North West England—Area-1: universal vitamin implementation-area (one of two), Area-2: targeted implementation-area—closely located, both in the most deprived fifth of local authorities, with similar life expectancies and childhood obesity, but Area-2 had a larger and younger population.

Governance involved:

—the University of Liverpool’s Faculty of Health and Life Sciences being Sponsor *UoL000752* for administration and management, as per Health Research Authority [29];
—National Research Ethics Service (NRES) (Proportionate Review Sub-Committee, East Midlands–Derby, ref. no. 11/EM/0362);
—three local research and development (R&D) committees.

### Quantifying Healthy Start take-up

Basic descriptive epidemiology compared take-up between English regions and between the North West primary care trust (PCT) areas, which included between universal (*n* = 2) and targeted (*n* = 22) implementation-areas (Additional file 2)**.** Analysis used Chi-squared test (IBM-SPSS v20).

### Qualitative

#### Preparation and approach

Quantitative findings and pilot interviews (two commissioners, two midwives, one mother) influenced topic-guide and participant information-sheet development. The topic-guide [30] allowed flexible semi-structured interviews, including in-depth exploration of unanticipated topics.

One author (MM) interviewed the all-female participants on NHS or local authority premises or via telephone (February–September 2012), with written or electronic consent, highlighting the information-sheet, confidentiality, and ability to withdraw without detriment. Data saturation [31] was progressively sought at the level of individual interviews and the overall dataset. Voice-recordings were deleted after transcripts were checked.

Tong et al.’s checklist [32] guided reporting, implicitly or explicitly. Regarding two of the 32 items though, there was no participant-checking of transcripts or findings.

#### Interviews

*Mothers’ (n = 25: 11 universal area; 14 targeted area)* interviews involved a diverse purposive sample of *potentially eligible Healthy Start* beneficiaries, whether entitled (application accepted) or not, i.e. English-speaking mother of an under-4-year-old, attending one of six children’s centres (universal area: two; targeted area: four) but using no other criteria.

The six children’s centre managers facilitated recruitment. MM briefed mothers (identified by midwife interviewees or visiting mothers) by telephone or in infant-toddler groups. Three mothers refused interviews. Participants confirmed if they were Healthy Start-entitled or not.

*Health professionals (n = 11: 5 universal area; 6 targeted area)*: All local authority-employed health professionals working with Healthy Start vitamins participated. Midwives and health visitors (*n* = 8) mostly chose telephone-interview and health promotion officers (*n* = 3) chose face-to-face interview at the centre.

*Commissioners and national HSIU staff (n = 6)*: Interviews were with the Healthy Start commissioners (n = 3) from Area-1 and Area-2 and, giving a second perspective, from the only other North West universal implementation-area (Area-3) plus the three national HSIU staff for context. No-one declined.

#### Qualitative data analysis

Qualitative analysis of anonymised transcripts used QRS-NVivo v9.2 and followed the tenets of Ritchie and Spencer’s [33] framework approach [34], particularly its ‘diagnostic’ (why things are so) and ‘evaluative’ (what affects effectiveness) aspects. *Familiarization* with the raw data (immersion) and field-notes identified emergent and recurrent themes [35]. Abstracting, conceptualizing, discussing between co-authors, and coding five transcripts generated and refined iteratively a *thematic framework* of deductive (from topic-guide) and inductive components. Full analysis included *indexing* and *charting* (case-charts, theme-charts). *Mapping* sought associations, wide-ranging experience, and exceptions.

### Observations of practice

At each children’s centre, MM gained permission to review the content of the locked vitamin cupboard (Additional file 2). MM checked availability and cost of similar vitamins by walking to the nearest pharmacy.

## Results

### Quantitative

Take-up of food vouchers exceeded vitamin vouchers considerably, and vitamin take-up remained very low, e.g. in the 3.5 years up to and including the immediate post-interview phase, in the North West:

- Food voucher take-up decreased marginally from 80.9 to 79.3% but remained moderately high.
- Women’s and children’s vitamin take-up increased slightly, respectively, from 0.2 to 4.3% and 0.5 to 2.5%, but remained very low (Additional file 2).

Nevertheless, food and vitamin voucher take-up appeared higher in the universal versus targeted areas of the North West, and the comparison was statistically significant, e.g.:

- Children’s vitamins: 6.3% (376/5961) versus 1.8% (1736/94563) (*p* < 0.0001, χ^2^ = 545.2) (Additional file 2).

### Qualitative

This section uses the following abbreviations:

*(N)EM = (non-)entitled mother; HP = health professional; C = commissioner; DH=Department of Health; universal = U, targeted = T.*

The qualitative component explored why these observed differences in take-up arose.

#### Why was food voucher take-up more than for vitamin vouchers?

*Mothers* explained higher food voucher take-up mostly in terms of awareness, accessibility, and acceptability. Those vouchers were conspicuous in the letter and straightforward (“*brilliant*” *EM18-U*) to use:

*“[The letter] has it all there on the bottom. It told you, you can go to any supermarket and stuff, [ …] if you go to your corner shop you can use them. If you go to Tesco’s, Asda, Sainsbury’s…” EM26-T*

Despite misunderstanding about participating shops *(“It’s only like the Asda or… that’s the only place I really know that takes them” EM27-T*), food vouchers’ monetary value was crucial:

*“…any money that they can get off the shop is great…, but I think if people don’t take vitamins anyway then they’re not likely to go and pick them up at a children’s centre; it’s another thing to remember…” EM40-U*

The monetary value seemed inadequate though, because *“with a £3.10 voucher you are having to pay up three of them together to cover the cost of one powdered milk… normally [needing] one a week…” EM10-U.*

*Health professionals* also mostly explained higher food voucher take-up in terms of awareness, accessibility, and acceptability. Echoing mothers, they mentioned better visibility (“*much more noticeable” HP04-U*) in the letter plus practical and monetary value:

*“…she’s £3.10 better off a week… she can go to the shop and actually use it for… fruit and veg and milk so there… whereas for the [vitamin] vouchers she has to go to a health centre, doesn’t she, and pick them up, or a community place [e.g. children’s centre or clinic]…” HP04-U*

*Commissioners* explained higher food voucher take-up with reasons for low vitamin take-up.

#### Why was vitamin voucher take-up so low?

*Mothers* explained low vitamin take-up in terms of suboptimal awareness (and attitude) (Table 1), accessibility, acceptability, and adequacy of supply. Mothers in both areas were unclear on eligibility, overlooked receiving the national vitamin vouchers, and mentioned ‘missing out’:

*“I have seen all these pictures and I thought ‘I wonder what that is?’ and then, when they [health professionals] did actually make me aware, I didn’t realise it is from when you are 12 weeks pregnant… So, I had missed out on all that time, my whole pregnancy, [through] a lack of communication.” EM13-T*

Entitled and non-entitled mothers from both areas were also unaware of vitamin benefits, feeling poorly informed by health professionals (*EM06-U,* Table 1). A frustrated mother wondered *“What was the point!”* and stopped seeking further vitamins, feeling *“put-off”,* after feeling dismissed with, *“Alright, here, take these” NEM16-T* (also Table 1).

**Table 1:** Why was vitamin voucher take-up so low? Lack of ‘awareness’ theme: Illustrative quotations

**Mothers (Universal): Own lack of awareness of the vitamin scheme or receiving vouchers** *“I got the food ones, but I never got the vitamin vouchers, and this has been going on for two years! All I ever get is the food vouchers and a letter.” EM10-U* *“…couldn’t remember if I had received any, ‘cause it doesn’t look like a voucher at the top. …by the time a friend told me about it, I was like ‘oh gosh, I will have to use it’, and then they stopped coming [because she returned to work] so I missed out.” EM18-U* *“No, I only just started getting the vitamin tablets with my second child; …because no one told me.” EM06-U* **Mothers (Targeted): Own lack of awareness of where to use vouchers and feeling poorly advised** *“…I’ve always seen the vouchers and thought… ‘oh I will have to find out where you go…’, but I’ve never actually followed it through.” EM30-T* *“Yeah [I noticed the vitamin voucher]… but it didn’t say where you get them from or how…” EM25-T* *“No, no one has spoken to me about vitamins. ...and I did actually go and ask my GP because… I was slightly overweight, so… […] she basically just told me to join Weight Watchers, and I wasn’t given any other advice at all.” NEM16-T* **Health professional (Universal): Mothers’ lack of awareness of vitamin vouchers** *“I wonder how much notice people take of it because it is just an add-on on the form [letter] really” HP04-U* **Health professional (Targeted): Mothers’ lack of awareness of vitamin/voucher importance** When *HP14-T* highlighted the voucher: *“…they say ‘oh yeah I get that all the time’ and when you explain to them that the take-up of the vitamins is really low and that you can get them from here [for free] they are shocked and they go ‘OK’ but they sometimes still don’t come [to the centre] and get them. I don’t think they understand the relevance or the importance...” HP14-T* **Commissioner (Universal): Mothers’ lack of awareness of vitamin importance** *“a lot… don’t particularly believe in the need” C03-U* **Commissioner (Targeted): Health professionals’ lack of awareness of who decided eligibility** *“I met with health professionals at the hospital… [but] instead of… [discussing] Healthy Start and the vitamins, and getting them signed up, they just said, well, we’re not doing this, because we’ll be making a judgement about the women. They missed the point completely! […] The [HSIU] has the information and will make the decision about eligibility’.” C01-T*	

From interviews in 2012 with potentially eligible mothers, health professionals, and commissioners about Healthy Start (in a universal and a targeted area in North West England)

(N)EM = (non-)entitled mother; HP = health professional; C = commissioner; universal = U, targeted = T

*HSIU* = Healthy Start Issuing Unit

The letter did not say where to obtain the vitamins and mothers did not recall being told, particularly in the targeted area: *“I now know it was my midwife who should have told me from day one!”* EM13-T. Some entitled mothers were aware of receiving the vitamin vouchers but did not use them (*EM30-T, EM25-T,* Table 1).

Dissatisfaction with taste (*“too chalky” EM38-U*) or palatability *(“I just throw up” EM06-U*) also stopped vitamin use. Complicated administrative processes and perceived unfairness also caused dissatisfaction:

*“I have a friend who has just turned 18 [in January] and she’s pregnant. She can’t get her vouchers until she claims child tax credits. She can’t claim child tax credits because they’ve just changed the rules and her mum has to claim child benefit for her until September… So, the government is expecting her to live, and her baby, to live off £20…* [sighs]*…” EM38-U*

For some entitled mothers in the targeted area, poor vitamin supply discouraged continued use: *“[children’s centres] just never have them.” EM26-T*. Futile searching for children’s vitamin drops was typical: *“everywhere I ask they go ‘we haven’t got them in’, like in the children’s centre…” EM27-T.*

*Health professionals* from both areas also explained low vitamin take-up in terms of suboptimal awareness (of mothers and health professionals), accessibility, attention, agency*,* and adequacy of supply (but not acceptability).

The vitamin voucher ‘hidden in plain sight’ was a substantial barrier (*HP04-U,* Table 1)*.* One health professional attributed poor maternal awareness of vitamin benefits to lower socio-economic status and education (*HP14-T*, Table 1).

From universal and particularly targeted areas, health professionals lacked knowledge (e.g. which vitamins; from where; or whether suitable for special diets). Some health professionals knew of colleagues withholding the vitamins through misunderstanding the constituents (mistakenly *“thought vitamin A was in the pregnant woman’s [vitamin tablets]*” *HP02-T*), blaming this on suboptimal training. Vitamin vouchers were not a priority in consultations *(“we midwives can be quite precious about our time”* HP05-U) or training:

*“They probably said ‘oh here you are, you can give these healthy vitamins’.” HP36-T*

Some health professionals (mainly from targeted area) did not know where mothers could redeem vitamin vouchers: *“…there should be a list of addresses…” HP24-T.* Some were unclear about the administrative processes:

*“I had absolutely no idea until last Thursday, that, when you’ve had your baby, in order to get the vitamin drops, you have to let [HSIU] know…” HP02-T*

Health professionals sometimes forgot or attended poorly to discussing the vitamins, particularly blaming inadequate training and perinatal staffing and a crammed consultation:

*“We definitely need to cover reducing the risk of cot death… We then talk about immunisations, ask them to sign an intent form for the Child Health department for when the immunisations are due. We talk to them about development checks, their own health, any family history of anything, and we also talk about smoking, alcohol, diet, and smoke alarms, child benefits, and somewhere in there we have to fit in the vitamins! And that’s for a straightforward mum; some of the cases I come across in the community have safeguarding issues and the like.” HP05-U*

Nevertheless, no-one suggested improving communication between, for example, midwives, health visitors, and social workers to improve their individual and collective agency in the system. Staying connected with close colleagues was hard enough:

*“Even within your own team, […] even when you’re all working towards the same goal, you are working in silos to a degree…” HP08-T*

Complicated administration introduced delay, blocked access, and frustrated staff with the form-filling (chasing applications *“for a 97p bottle of vitamins” HP15-T*), ‘hidden’ vitamin vouchers, and poor supply:

*“…quite frequently I get people ringing me asking me for the Healthy Start number, because they haven’t heard [about their application]…” HP04-U*

To avoid ‘red tape’, health professionals thought that they should personally hand mothers the vitamins, particularly if vulnerable (*“instead of the mothers having to go somewhere else” HP04-U*, i.e. to another centre):

*“…then they have to ‘re-register’*^†^ *once the baby is born so it’s… a lot of red tape and forms…” HP15-T [*^†^*This can be by telephone though.]*

*“I am working with [a family in a complex situation] and she has had difficulties of obtaining the vitamins, due to [moving] a few times, and she’ll have been backwards and forwards with… in care, and the child is nearly 3 and could have really done with them, and all of the red tape has completely put her off… I have even rang the Department of Health and they can’t speak to me about it, because it’s her…” HP14-T*

Illustrating lack of agency, some health professionals in the targeted area felt frustrated and powerless at being unable to give vitamins to non-entitled families requesting them:

*“…we had a family recently… She was from an ethnic minority and there were definitely some signs [of vitamin D deficiency], and I did recommend that she go to a doctor… she didn’t do that but… …She goes, ‘please just let me buy them’… ‘I can’t because there is a lot of red tape, again, surrounding the purchase of them’. She understood the need [but] all I could do was recommend… her to… find something similar, which was a big, big deal for her.” HP15-T*

*Commissioners* from both areas highlighted aspects of awareness, accessibility, agency, and adequacy of supply, but also accountability. Besides mothers’ lack of vitamin awareness (*C03-U,* Table 1), commissioners appeared surprised and disappointed at poorly-informed health professionals, particularly in the targeted area. Some health professionals offered no vitamins as they believed mistakenly that:- they had to judge maternal socio-economic status; the vitamins for pregnant women inappropriately contained vitamin A (present in children’s vitamin drops *only*); or the vitamins were unsuitable for special diets. One commissioner highlighted health professionals’ lack of awareness of decision-making about eligibility (*C01-T,* Table 1).

Commissioners also cited poorly visible vitamin vouchers. One commissioner who had worked in the universal area since Healthy Start began highlighted improvements though:

*“Healthy Start put a lot of things in those letter packages to people. And originally […] it was one line [about vitamins] on the letter and the writing was very small, but Healthy Start improved the look of the voucher; […] but it still wasn’t as big as the food voucher.” C03-U*

It was costly for mothers to telephone the HSIU to ask questions or to declare the birth to obtain children’s vitamin drops (albeit simpler than originally having to re-apply):

*“women can [now] just make a phone call and say, ‘I’ve had my baby’ […], …but it’s complicated… […] often, women who are in low-income households do not have a landline within their house, and they were using mobiles, […] on premium-rate numbers…” C01-T*

Underfunding of local Healthy Start vitamin programmes affected both accessibility and supply, with complicated administrative processes. The commissioners described how the HSIU would fulfil their vitamin orders via the NHS ‘supply chain’ (distribution service), which delivered only to NHS estates. Without extra funding, the commissioners were responsible for local distributors (e.g. children’s centres) receiving vitamins. Delays meant out-of-date vitamins. Commissioners relied on the goodwill of other local distributors to overcome national governance requirements:

*“Through seeking the help of Estates [in the commissioning organization], we identified a local mailing van, like the NHS mail-van that goes from clinic to clinic. We identified one that goes from children’s centre to children’s centre. This really made distribution of the vitamins simple.” C03-U*

*“Estates were involved in distribution of the vitamins ‘cause there was a lot of governance issues because we had NHS providing to the local authority […]. …but the way that we worked, it worked absolutely fabulously […] …Department of Health kept on saying to us – no you can’t do this because [of] governance issues, whilst it worked for us.” C22-U*

Within commissioning, their ‘collective agency’ was over-reliant on goodwill to ensure vitamin distribution via hospitals and rather resistant general practices: *“how much are you going to give me for doing this?” C01-T*. Logistics were tricky:

*“…[NHS] people… say, ‘hang on a minute, you are asking me to do something that’s out of my job description!’ And […] the Department of Health had never thought this through properly… […] This was all supposed to be done out of goodwill!” C01-T*

Children’s vitamin drops were a medicine (not a supplement), requiring local pharmaceutical approval. One commissioner worked around this with local Medicines Management:

*“…we had one of their managers… arranged… approval for us to order through her, so everything was purchased up front, everything was distributed from Medicines Management, and then the accountant […] put [that] in as a return.” C01-T*

Commissioners from both areas were frustrated at the ad hoc vitamin supply affecting take-up:

*“…health visitors were reluctant to tell somebody to go and get something that they thought was highly likely not to be there for them. So, even when you had them stocked, they’d end up in the bin, because no one claimed them; we were paying to throw vitamins in the bin…” C03-U*

Commissioners believed that improved vitamin take-up required more accountability. One commissioner was astonished that the HSIU did not *“want to know if the vitamins actually got to mothers; all they want is purchase data*” *C01-T*. Regular steering-group meetings in both areas encouraged accountability for vitamin distribution and take-up. In the targeted area, local authority staff in children’s centres appeared more engaged than NHS staff, possibly because local authority targets and inspections included Healthy Start vitamin performance:

*“Children’s centres […] have ‘OFSTED’ inspections… Distributing Healthy Start vitamins is another way that they can show that they’re being beneficial to the community…” C01-T*

The HSIU was unconvinced about challenging ‘nought returns’: “*I think it would be unheard of for a government department to legally challenge another bit of the same public sector*” *DH44-HSIU.* One commissioner from the universal area believed that ‘nought returns’ reflected that *“it is too costly for them to [file the return]” C03-U.*

#### Why might vitamin voucher take-up be more in the universal area?

Higher vitamin take-up (albeit still low) in the universal area related to awareness (staff), accessibility, attention (priority), and adequacy of supply (Table 2).

**Table 2:** Why might vitamin voucher take-up be more in the universal area? llustrative quotations of themes

**Mothers (Targeted): Accessibility and Adequacy of supply** *“I try [to use the vitamin vouchers], if you can get them for free, you’ve seen I’ve got the voucher right here… but… I don’t know whether it’s because of the cutbacks or whether they’ve just stopped sending them, but the [children’s] centres where I go [to] ask for them—they just never have them.” EM26-T* *“There was nowhere really to get them. Every time I’d ask in the doctors’ they said, ‘see your midwife’, and the midwife told me to look on the internet, but I haven’t got any internet at home… I have never ever got the vitamins because I don’t know where to get them from or anything…” EM27-T* **Health professionals (Universal): Awareness and Accessibility** *“…available in every children’s centre so that’s… six… within a radius of about ten miles? So they’re quite freely available […] from the receptionist…” HP05-U.* *“Now locally […] [mothers are] given a form to come and get vitamins… from the children’s centre… […] Often I will give one of those forms to everybody because it means they can go and get them straight away because Healthy Start when you apply [takes ages] to come through.” HP04-U* **Commissioner (Universal): Accessibility and Adequacy of supply** When the universal area had been a targeted area: *“The clinic staff [receptionists] outside here, because they were very rarely asked for [the vitamins], or would forget [to ask mothers for the voucher], and they would go out of date… And managers would just stop stocking them… they’d just fall off the agenda.” C03-U* Overclaiming did *not* explain higher vitamin take-up. Reimbursement claims related only to entitled mothers: *“We keep a spreadsheet …from each children’s centre… mark E for eligible or L for local…” C03-U* [Mothers could only use either the locally produced or national voucher.] **Commissioner (Targeted): Attention (prioritizing)** Key stakeholders paid insufficient attention to improving vitamin take-up. A working group lacked midwife and GP engagement and *“we failed miserably” C01-T* to engage an accountant to help file HSIU returns. That commissioner also wanted more active listening from: *“[the Department of Health] …get people from different parts of the country, sit down and talk to them and say, OK, what, what are our barriers?” C01-T*	

From interviews in 2012 with potentially eligible mothers, health professionals, and commissioners about Healthy Start (in a universal and a targeted area in North West England)

EM = entitled mother; HP = health professional; C = commissioner; universal = U, targeted = T

*HSIU =* Healthy Start Issuing Unit

*Mothers* from the universal area reported that they could exchange vouchers for vitamin tablets or drops in several places (*“I can get them here [children’s centre]; there’s lot of places I could get them” EM09-U*), and the process appeared easy and immediate *(“they just gave me this… yellow card* [a local card]*, and each time you come you have to have it signed” NEM19-U; “went to a weaning group and they told us… [and] we got them [there]” NEM12-U*). This accessibility and adequacy of supply contrasted with mothers’ frustration in the targeted area at the ‘mystery’ of supply.

*Health professionals* in the universal area seemed more knowledgeable about vitamin access, which they considered to be timely but might improve *“if midwives actually had them…” HP04-U* to provide at the ‘booking-in’ (first) consultation.

*Commissioners* in the universal area considered that offering Healthy Start vitamins to all women raised staff awareness because previously, when their Healthy Start was targeted, access and adequacy of supply suffered.

## Discussion

This study has shown that the national vitamin vouchers were hidden in plain sight, evading busy mothers and staff caught in an overcomplicated system. Poor Healthy Start vitamin take-up from the statutory scheme was linked to precarious, overcomplicated procedures that confused, frustrated, or escaped its main stakeholders. Scheme success relied on underfunded children’s centres, organizational goodwill, and the resilience of participating families. Food and vitamin voucher take-up was significantly higher in the universal administration-areas of the North West compared with the remaining areas. It seems likely that barriers (including stigma known to be associated with parental participation in targeted children’s services [36] such as in children’s centres) decrease when the vitamin provision becomes universal. The old public health adage that *“public health is everyone’s business”*, which has recently been a guiding principle for public health teams in the pandemic [37], should underpin universal Healthy Start vitamin supplementation and make it easier for health professionals to implement.

Studying mothers’, health professionals’, and commissioners’ perceptions about Healthy Start take-up suggested that take-up was consistently much higher for food than vitamin vouchers because the food vouchers had clearer presentation and messaging and had practical and monetary (albeit low) value. Barriers to vitamin take-up included suboptimal awareness (and attitude) of mothers and staff, attention to its priority, accessibility, acceptability, and adequacy of supply.

All pregnant women in Scotland have qualified for free Healthy Start vitamins since April 2017 [38, 39]. Attempts to improve England’s scheme have continued, e.g. more obvious vitamin vouchers and the food voucher covering pulses and frozen vegetables, but the scheme remains broadly similar to when our study was undertaken in 2012. Digitizing vouchers [40] has been delayed but a pre-paid card replaces paper vouchers in 2022.

While the food voucher works better than a cash-equivalent benefit [9, 41], and its subversion [42] is probably uncommon [43], its monetary value has progressively lagged food costs [12, 25]. From April 2021, the weekly food voucher increased from £3.10p (since 2009) to £4.25p (matching Scotland) [44].

Low food voucher value might explain falling take-up over this last decade. By the beginning of 2020 and 2021, take-up was only 54 and 53% in England and 54 and 54% in the North West, respectively [28], i.e. down about one-quarter on 2012/13 (Quarter 1 (Q1): 70 and 73%, Additional file 2). Jessiman et al. found that midwife reminders about Healthy Start eligibility were patchy and beneficiaries easily fell off the scheme, especially by not reporting the birth [24]. Furthermore, health professionals may well progressively add well-intentioned edicts (to avoid fraud or non-viable pregnancies), which then delay the application process, and *“the fragile nature of improvement work*^**”**^ **[**45] ^**(p4)**^ hinders sustained remediation.

Meanwhile, continued very low vitamin take-up receives little attention, documented when individual public health teams publish quarterly HSIU [28] data. The HSIU website was displaying *food* voucher take-up data only but now none. The figures are stark. For example, Birmingham implements Healthy Start universally and has documented systematically a 5-year falling ‘vitamin take-up for eligible’ people from about 4 to < 1% (and for non-eligible from about 25% to about 10%) [46]. In 2020, one member of parliament highlighted Healthy Start dataset inadequacies [47], an underpublicized issue.

This study complements and extends findings from a similar era about frustrated staff trying to overcome improvement barriers [21, 23–26]. They encountered Heath Robinson-level [48] system complications. In our study though, health professionals in the universal area seemed more knowledgeable about vitamin access and devised local solutions to supply-chain barriers. Futile searching by mothers arose from suboptimal vitamin access and supply, similar to Jessiman et al. [24], particularly when targeted. Nevertheless, even a universal scheme commissioner felt like*, “we were paying to throw vitamins in the bin”*. Jessiman et al. [24] found that midwives engaged more directly in universal pilot-areas by issuing vitamins in-person with timely advice to embed the vitamins ‘habit’ [43] ^**(p77)**^.

This study contextualizes calls for universal implementation [23, 24, 49, 50]. This would only be cost-effective if covering all the target group *plus* all women planning pregnancy or below 10 weeks’ pregnant (reflecting folic acid impact), infants 0–6 months, and 4–5 year-olds [51].

Machell’s [52] policy analysis of how Healthy Start developed highlighted little meaningful convergence of the problem tackled for potential beneficiaries, the implementation policy, and the politics. Politics drove development not evidence about eligible beneficiaries’ food culture and access to food. In our study, mothers, i.e. Machell’s *“hidden participants”* [52] ^**(p24)**^ to policy-making, clearly articulated unmet need, despite the Government now describing Healthy Start as *“demand-led… not target driven”* [53] and apparently ignoring food and vitamin vouchers in its ‘The Best Start for Life’ vision [54]. If the vitamin scheme were to be truly ‘demand-led’, lessons from use of social marketing to tackle COVID-19 vaccine hesitancy might suggest using a ‘demand generation’ strategy [55]. While this might improve the Healthy Start brand and communication for families and health professionals, supply problems would still have to improve considerably. We found that even when some mothers expressed a need for meaningful access to Healthy Start vitamins, various barriers thwarted them, including some misinformation or misguided actions from health professionals.

### Strengths/limitations/implications

This study involved only two local administrative areas, however the dystopian experience of staff and 25 potentially eligible mothers converged from six children’s centres sampled specifically to compare a similarly deprived universal and targeted area. Jessiman et al. [24] likewise recruited successfully via children’s centres, but their 107 parents were from a single postcode in thirteen English administrative areas that were sampled on criteria other than universal versus targeted implementation. While we did not sample according to maternal education-level or number of children, this was consistent with other contemporaneous studies [24, 25] and would have been logistically difficult while already imposing on the goodwill of the children’s centres and families. Nearly all McFadden et al.’s [25] 109 (potential) beneficiaries’ views (including four males) came from participatory workshops and focus groups, but our study used semi-structured interviews for in-depth probing, like Jessiman et al. [24].

Our findings have illuminated this unfit-for-purpose vitamin-delivery system. Since the data were collected, child poverty has continued to grow and population nutrition to deteriorate, emphasizing the need for action. Crawley and Dodds [38] ^**(p7)**^ considered this to be:

*“a new era of child poverty and family food insecurity […] The reduction in the number of families eligible for Healthy Start appears incongruous against this backdrop of increasing hardship among low-income families.”*

They concluded that the scheme *“has not been consistently supported either nationally or locally”*^**(p69)**^ and benefits system changes have not undergone impact assessment for Healthy Start. They recommended commissioning *“a regular review of the effectiveness of the Healthy Start scheme in achieving its public health goals”*^**(p17)**^ and commissioning SACN or National Institute for Health and Care Excellence (NICE) to advise *“on reformulating the Healthy Start vitamins as primarily vitamin D supplements”*^**(p15)**^.

While it is a limitation that we are reporting data from several years ago, the findings are still highly relevant to today’s Healthy Start scheme. Since our own study, system improvements have been very few, small, and fragmented (e.g. food voucher value increasing modestly, belatedly, in 2021, and paper vouchers now becoming digital pre-paid cards). Meanwhile, progressive underfunding of children’s centres has compromised health improvement [56] and Healthy Start delivery. An early report from Haringey (London) suggested that a project to improve Healthy Start food voucher take-up during 2020 led to 22% more families receiving the vouchers. Nevertheless, a 39% increase in eligible families, whether due to the project or to the pandemic, gave a net decrease in take-up from 56 to 49% [57]. It is therefore important that our findings can be used to advocate for a better Healthy Start scheme overall.

Healthy Start has become news again though in 2021. Marcus Rashford (English Premier League footballer) has promoted voucher take-up in his healthy nutrition campaign for low-income families [58, 59], for whom determinants of ‘nutrition choices’ are more structural than lifestyle [60]. Additionally, the Health Secretary (England) conceded a legal challenge in June 2021 about a low-income mother who was awaiting settled UK status being deemed ineligible [61]. This prompted government commitment to reviewing the scheme to ensure that it is non-discriminatory.

Our study should be replicated elsewhere to contribute to such a review, updating on participants’ insights.

### Conclusion

*Substantive* Healthy Start reform in England (not just cosmetic tinkering) is long overdue. Meanwhile, childhood nutritional rickets continues in high-risk groups lacking intended Healthy Start vitamins [62]. Poverty-related food insecurity still requires much re-investment in early years’ services [1]. *“Meaningful engagement and co-production with people with lived experience of poverty*^**”**^ **[**63] ^**(p5)**^ would mean asking families how best to simplify vitamin take-up. Our study highlights that ‘policy, politics, and problem’ should be aligned to reach considerable unmet need.

## Supplementary Information

**Additional file 1: Supplementary Additional file 1.** Healthy Start vitamins--Sequential explanatory mixed-methods study design.pdf.

**Additional file 2:** **Supplementary Additional file 2.** Healthy Start vitamins--Quantitative--commentary and table.pdf.

## Data Availability

The qualitative datasets generated and analysed in this study are not publicly available due to data privacy reasons, as per ethics approval. The Results section presents the relevant interview data from written transcripts of participants’ audio-recordings, which were deleted post-transcription as per approval agreements. The corresponding author would answer questions about these data. The quantitative data were obtained and used with permission from the Healthy Start Issuing Unit. Such data requests should be directed to that Unit [28].

## Abbreviations

CMOs: UK chief medical officers
HSIU: Healthy Start Issuing Unit
NICE: National Institute for Health and Care Excellence
NRES: National Research Ethics Service
PCT: primary care trust
Q1: Quarter 1 (of data collection-year)
R&D: research and development
SACN: Scientific Advisory Committee on Nutrition
UK: United Kingdom
