# Supplementary material for: An exploration of the statutory Healthy Start vitamin supplementation scheme in North West England
Source: BMC Public Health. 2022 Feb 24;22:392. doi: 10.1186/s12889-022-12704-0 (PMC8869346; doi:10.1186/s12889-022-12704-0)
Supplement: Supplementary file 1 — Additional file 1: Supplementary Additional file 1. Healthy Start vitamins--Sequential explanatory mixed-methods study design.pdf. [file 12889_2022_12704_MOESM1_ESM.pdf]

Additional file 1: Sequential explanatory mixed-methods study of Healthy Start vitamin take-up in North West England, 2012

Quantitative<br>**Healthy Start Issuing Unit (HSIU) quarterly take-up data to set context**	informed →	Qualitative:<br>**42 semi-structured interviews to explore perceptions and experiences**
• North West England, English regions, and England<br>• % take-up of Healthy Start vouchers for food, women's vitamins, and children's vitamins around the time of the interviews		• North West England<br>• Mothers, health professionals, and commissioners<br>• About Healthy Start voucher take-up<br>• Explored reasons for the three main quantitative findings:<br>– Why was food voucher take-up more than for vitamin vouchers?<br>– Why was vitamin voucher take-up so low?<br>– Why might vitamin voucher take-up be more in the universal area?
