# Supplementary material for: An exploration of the statutory Healthy Start vitamin supplementation scheme in North West England
Source: BMC Public Health. 2022 Feb 24;22:392. doi: 10.1186/s12889-022-12704-0 (PMC8869346; doi:10.1186/s12889-022-12704-0)
Supplement: Supplementary file 2 — Additional file 2: Supplementary Additional file 2. Healthy Start vitamins--Quantitative--commentary and table.pdf. [file 12889_2022_12704_MOESM2_ESM.pdf]

Additional file 2: Healthy Start findings: Quantitative (and Observations): e-Commentary and e-Table

Expanded commentary (colour-coded to link to data in e-Table below): Take-up (%) of Healthy Start food, women's vitamin, and children's vitamin vouchers, by English region and North West areas in the lead-up to 2012 interviews of mothers, health professionals, and commissioners about Healthy Start voucher take-up (North West England)

Take-up of food vouchers exceeded vitamin vouchers considerably just before this interview-study, in all nine English regions (e-Table below).

Over the first quarters (e-Table below) of 2009/10–2012/13, for example, median voucher take-up was: England=79.7% for food, 2.4% for women's vitamins, 2.0% for children's vitamins; North West=80.9%, 3.2%, 2.1%, respectively.

While take-up of food vouchers decreased slightly and of women's and children's vitamins increased slightly, vitamin take-up remained very low just before this interview-study. Overall, between Q1-2009/10 and Q3-2012/13 (just after the interview-phase), food voucher take-up decreased from (e-Table below for initial percentages):

79.7% to 77.2% in England — 80.9% to 79.3% in North West

whereas women's and children's vitamin take-up increased, respectively, from:

0.3% to 6.6% in England — 0.2% to 4.3% in North West

0.7% to 3.3% in England — 0.5% to 2.5% in North West

Take-up of food and vitamin vouchers appeared higher in the universal versus the targeted areas of the North West, being significantly higher in the two universal areas combined than in the targeted areas combined, e.g. in Q1-2010/11:

Food: 83.7% (4,401/5,258) versus 81.6% (69,685/85,404) ($p<0.0001$, $\chi^2=14.7$)

Women's vitamins: 11.0% (221/2,008) versus 3.0% (961/31,864) ($p<0.0001$, $\chi^2=358.1$)

Children's vitamins: 6.3% (376/5,961) versus 1.8% (1,736/94,563) ($p<0.0001$, $\chi^2=545.2$)

In the two interview-areas: Universal Area-1 take-up of women's and children's vitamin vouchers exceeded that for targeted Area-2 in Q1-2011/12 and Q1-2012/13, i.e. before and after the interview-period (e-Table below).

Vitamins in locked cupboards: Observations: In the targeted area, one centre had no vitamins (awaiting delivery for over two months) and the only centre with a cupboard-opening protocol was the best stocked. In the universal area, both centres had a protocol; one had no children's vitamins. Overall, vitamin supplies were minimal in the other centres.

At the nearest pharmacy to each centre (10-14 minutes' walk), the cheapest children's and women's vitamins were 3-4 times and 17-43 times the daily price of the Healthy Start vitamins, respectively.

e-Table: Take-up (%) of Healthy Start food, women's vitamin, and children's vitamin vouchers, by English region and North West areas, on one day in a 4-week cycle each quarter-year (Q): Q1 2009/10–Q1 2010/11 and Q1 2011/12 and Q1 2012/13

Vouchers for: F=food W=women's vitamins C=children's vitamin drops		Potential no.† to be claimed (2009/10 Q2)	Percentage 2009/10 *4-week cycle-no.* Q1-*81*	Q2-*84*	Q3-*86*	Q4-*89*	2010/11 Q1-*94*	2011/12 Q1	2012/13 Q1
East Midlands	F	*38,508*	79.9	80.1	80.2	80.4	80.6	80.2	72.6
	W	*14,068*	0.4	0.3	0.7	1.6	1.5	4.5	7.3
	C	*52,470*	0.8	0.2	1.1	1.7	1.3	2.6	3.2
East of England	F	*39,812*	75.1	75.0	75.1	75.2	75.4	75.1	68.5
	W	*14,604*	0.9	0.1	1.6	3.7	2.0	2.5	9.0
	C	*54,470*	0.9	<0.1	2.0	2.0	1.6	2.1	5.1
London	F	*94,266*	80.7	80.4	80.1	80.1	79.8	78.8	65.3
	W	*34,741*	0.2	<0.1	0.3	0.4	0.8	2.0	7.5
	C	*129,574*	0.6	0.3	0.6	1.1	1.3	2.2	4.0
North East	F	*29,667*	84.2	84.4	84.4	84.5	85.3	85.0	77.3
	W	*10,851*	<0.1	0.1	0.1	0.6	0.8	1.1	2.2
	C	*40,470*	0.1	0.2	0.2	0.7	0.7	0.6	1.3
North West	F	*72,130*	80.9	81.0	81.0	81.1	81.7	80.9	72.5
n=24 areas	W	*26,368*	0.2	1.5		1.9	3.5	2.8	4.5
	C	*98,347*	0.5	0.7	1.1	1.7	2.1	2.1	2.7
**Area-1-studied**: Universal	F			82.2	81.9	82.0	81.2	80.3	75.1
	W			---	1.1	3.4	6.9	8.7	15.5
	C			---	1.9	5.3	6.2	10.5	6.6
Area-3: Universal	F			85.1	84.8	85.3	86.5	84.4	68.5
	W			2.9	7.5	8.9	15.4	6.6	6.6
	C			2.8	4.5	5.4	6.4	7.3	8.5
**Area-2-studied**: Targeted	F			83.3	83.3	82.7	83.1	82.5	78.3
	W			5.9	2.3	4.8	14.7	7.1	3.4
	C			1.6	1.7	3.7	8.4	4.7	1.9
**Area-2 & 4→24**: Targeted	F			80.8	80.8	80.9	81.6		
	W			1.5	1.1	1.7	3.0		
	C			0.6	1.0	1.5	1.8		
South East	F	*53,030*	74.6	74.9	74.8	74.6	75.0	74.7	67.7
	W	*19,421*	0.2	0.2	0.2	0.5	1.8	2.2	5.5
	C	*72,435*	0.3	0.2	0.4	0.8	2.2	1.3	2.9
South West	F	*34,387*	77.8	78.0	78.1	77.8	78.2	77.5	70.6
	W	*12,515*	0.5	0.3	0.9	1.5	0.6	2.1	2.7
	C	*46,676*	0.4	0.5	1.0	1.5	0.7	0.5	1.6
West Midlands	F	*62,054*	82.2	82.1	82.3	82.3	82.9	82.8	72.7
	W	*22,628*	0.5	1.9	1.5	2.7	4.5	4.0	13.2
	C	*84,395*	2.0	1.1	1.9	2.1	4.1	2.8	6.2
Yorkshire & the Humber	F	*53,121*	81.5	81.8	81.4	81.9	82.4	82.1	72.6
	W	*19,287*	0.1	0.1	1.0	1.6	1.1	2.8	4.2
	C	*71,935*	0.4	0.1	1.7	2.7	1.7	2.0	2.3
England (total)††	F	*476,975*	79.7	79.8	79.7	79.7	80.1	79.6	70.4
	W	*174,482*	0.3	0.6	0.9	1.6	2.0	2.7	6.6
	C	*650,772*	0.7	0.4	1.1	1.6	1.9	2.0	3.5

At that time, earliest voucher take-up data made available from HSIU were Q1–2009/10 (April 2009). Data received changed over time: Q1-2009/10 to Q2-2010/11: numbers and %; Q3-10/11 to Q3-12/13: % only. *Several data-columns are suppressed above to de-clutter presentation.*

--- denotes: Local administrative area submitted no vitamin claim for reimbursement ▒*denotes: Raw data from HSIU were unavailable for calculation*

† Weekly food voucher(s) for: pregnant women=1; 1–4-year-olds=1; infants=2 8-weekly vitamin voucher for: women's bottle; children's bottle

††*England total is not the exact sum of the regions due to rounding.* *Department of Health HSIU (Healthy Start Issuing Unit) database*

Additional file 2
